# Genitourinary Syndrome of Menopause among Postmenopausal Women in a Tertiary Care Centre: A Descriptive Cross-sectional Study

**DOI:** 10.31729/jnma.7237

**Published:** 2022-02-28

**Authors:** Neebha Ojha, Kesang Diki Bista, Sunita Bajracharya, Neeta Katuwal

**Affiliations:** 1 Department of Obstetrics and Gynecology, Tribhuvan University Teaching Hospital, Institute of Medicine, Kathmandu, Nepal

**Keywords:** *atrophy*, *genital*, *Nepal*, *postmenopause*

## Abstract

**Introduction:**

Genitourinary syndrome of menopause incorporates vulvovaginal and lower urinary tract symptoms related to estrogen deficiency which affects more than half of postmenopausal women. This study aimed to find out the prevalence of genitourinary syndrome of menopause among postmenopausal women attending a tertiary care center.

**Methods:**

A descriptive cross-sectional study was conducted at the Department of Obstetrics and Gynaecology of tertiary care hospital from October 2019 to April 2021 after obtaining ethical approval from the Institutional Review Committee (Reference number: 452 6-11). A convenience sampling method was used among postmenopausal women aged 40-75 years attending gynaecology outpatient departments. The information was obtained by interview and examination. Analysis was done using the Statistical Package for the Social Sciences version 20. Point estimate at 95% Confidence Interval was calculated along with frequency and proportion for binary data.

**Results:**

Among the 385 postmenopausal women, 285 (74.02%) (69.63-78.40 at 95% Confidence Interval) had a diagnosis of genitourinary syndrome of menopause. The mean age of menopause was 48.2±3.6years. The most common symptom and sign were vaginal dryness in 223 (78.2%) and decreased moisture 200 (70.2%). Regarding the impact of genitourinary syndrome of menopause on quality of life, it least affected the activities of daily living and mostly on sexual functioning.

**Conclusions:**

The study showed three-fourth of postmenopausal women suffered from genitourinary syndrome of menopause which is similar to the previous studies done in other countries, in postmenopausal women. Therefore, understanding genitourinary syndrome of menopause is vital, and assuring women those treatments are available to relieve symptoms may improve their quality of life.

## INTRODUCTION

Menopause is associated with a variety of problems of which genitourinary problems are one of the commonest symptoms, affecting more than half of the women of menopausal age.^1-3^ In 2014, the new term Genitourinary Syndrome of Menopause (GSM) was introduced by the International Society for the Study of Women's Sexual Health and the North American Menopause Society.^4^

Most women feel awkward discussing vaginal and vulvar health. Even in countries like Canada, the United Kingdom, and Finland, 77% of respondents believed women were uncomfortable discussing vaginal atrophy and 42% did not know that local treatment was available.^5^ Therefore knowing and understanding GSM is vital in improving woman's health during menopausal years and assure the woman those treatments are available and can be taken to relieve symptoms.

The study aimed to find out the prevalence of GSM among postmenopausal women attending gynecology outpatient of a tertiary care center.

## METHODS

This is a descriptive cross-sectional study, which was conducted at the Department of Obstetrics and Gynaecology of Tribhuvan University Teaching Hospital, Kathmandu, Nepal from October 2019 to April 2021. Ethical approval was taken from the Institutional Review Committee (Reference number: 452 6-11). Postmenopausal women aged 40-75 years attending gynecology out-patient department for check-up were taken for the study. They were excluded if there was a history of hormonal replacement therapy, alcohol or drug abuse, or had a major psychiatric illness. Vulvovaginal conditions due to other pathologies like infections, irritants, allergies, dermatosis, and malignancies were excluded by local examination. Convenience sampling method was used.

Sample size was calculated using the following formula:

n = Z^2^ × p × q / e^2^

  = (1.96)^2^ × 0.50 × (1-0.50) / (0.05)^2^

  = 385

Where,

n = minimum required sample size
Z = 1.96 at 95% Confidence Interval (CI)
p = prevalence taken as 50% for maximum sample size
q = 1-p
e = margin of error, 5%

We have included 385 women meeting the eligibility criteria. The information was collected by interview regarding the age, marital status, ethnicity, occupation, educational status, and the duration since menopause. In the case of surgical menopause, confirmed cases of total hysterectomy with bilateral oophorectomy were taken for the study. History regarding the presence of genitourinary symptoms and gynaecological examination for the presence or absence of vulvovaginal signs were noted. Diagnosis of Genitourinary Syndrome of Menopause (GSM) was made based on signs and symptoms developed by the International Society for the Study of Women's Sexual Health and the North American Menopause Society (If at least two symptoms or one symptom and one sign are present).^4,6^

The impact of GSM on quality of life was assessed by using Day-to-Day Impact of the Vaginal Ageing (DIVA) questionnaire.^7^ This questionnaire was asked to the women who had at least one vaginal symptom. The questionnaire consists of 23 questions and focuses on four domains i.e., activities of daily living, emotional wellbeing, sexual functioning and selfconcept, and body image. For questions regarding sexual functioning, two separate scales were used: a short version for all women and a longer version only for those who were sexually active within the last one month. Each item was scored from 0 to 4 for the severity of the symptoms. Questions regarding the Day-to-Day Vaginal Aging (DIVA) had been translated into Nepalese version for better understanding to the patient.

The collected data were entered and analysed in the Statistical Package for the Social Sciences version 20. Descriptive statistics were used and the categorical variables were interpreted by frequencies and percentages. Continuous variables were summarized into means with standard deviations. Point estimate at 95% Confidence Interval was calculated along with frequency and proportion for binary data.

## RESULTS

Among the 385 postmenopausal women, 285 (74.02%) (69.63-78.40 at 95% Confidence Interval) had a diagnosis of Genitourinary Syndrome of Menopause (GSM).

Among women with GSM, 121 (42.5%) were sexually active. Vaginal dryness was the most common symptom affecting 223 (78.2%). In the sexually active postmenopausal women with GSM, decreased lubrication affected 89 (71.1%) and pain during contact in 75 (61.2%) women. The most common signs were decreased moisture 200 (70.2%) and decreased elasticity in the vagina 173 (60.7%). Among the urinary symptoms, 137 (48.1%) had dysuria and 156 (54.7%) had urinary frequency/urgency (Table 1).

**Table 1:** Urogenital symptoms and signs in postmenopausal women with genitourinary syndrome of menopause (n= 285).

Symptoms	n (%)
Genital dryness	223 (78.2)
Decreased lubrication with sexual activity^*^	86 (71.1)
Discomfort or pain with sexual activity^*^	74 (61.2)
Decreased arousal, orgasm desire^*^	70 (57.9)
Irritation/Burning/Itching of the vulva or vagina	154 (54.0)
Dysuria	137 (48.1)
Urinary frequency/urgency	156 (54.7)
**Signs**	
Decrease moisture	200 (70.2)
Decreased elasticity	173 (60.7)
Labia minora resorption	166 (58.2)
Pallor/erythema	101 (35.4)
Loss of vaginal rugae	133 (46.7)
Tissue fragility/petechiae	71 (25.2)
Loss of hymenal remnants	126 (44.2)
Introital retraction	64 (22.5)
Prominence of urethral meatus	65 (22.9)

* calculated in women with sexual activity within last one month with GSM (n= 121)

Among women with menopause duration of ≤5 years 65 (60.2%) were sexually active while in women with menopause of >5 years 56 (31.6%) were sexually active. Most of the urogenital symptoms and signs were more frequent in women who were menopausal for 5 years or more in comparison to the lesser duration of menopause (Table 2).

**Table 2:** Urogenital symptoms and signs according to the duration since menopause in women with GSM (n= 285).

Duration since menopause		
	≤5 years (n= 108) n (%)	>5 years (n= 177) n (%)
Symptoms		
Genital dryness	82 (75.9)	141 (79.7)
Decreased lubrication with sexual activity^*^	49 (75.4)	37 (66.1)
Discomfort or pain with sexual activity^*^	41 (63.1)	33 (58.9)
Decreased arousal, orgasm desire^*^	38 (58.5)	32 (57.1)
Irritation/Burning/Itching of the vulva or vagina	57 (52.8)	97 (54.8)
Dysuria	52 (48.1)	85 (48.0)
Urinary frequency/urgency	53 (49.1)	103 (58.2)
Signs		
Decrease moisture	66 (61.1)	134 (75.7)
Decreased elasticity	59 (54.6)	114 (64.4)
Labia minora resorption	47 (43.5)	119 (67.2)
Pallor/erythema	28 (25.9)	73 (41.2)
Loss of vaginal rugae	40 (37.0)	93 (52.5)
Tissue fragility/petechiae	21 (19.4)	50 (28.2)
Loss of hymenal remnants	38 (35.2)	88 (49.7)
Introital retraction	18 (16.7)	46 (26.0)
Prominence of urethral meatus	20 (18.5)	45 (25.6)

* calculated in women with sexual activity within last one month with GSM (n= 121)

The vaginal symptoms had the least impact on the activity of daily living (mean score= 0.6) and some impact on emotional well-being (mean score= 1.0) and self-concept and body image (mean score= 1.2). The highest impact was seen in the sexual functioning who were not sexually active (mean score= 1.8). All the mean scores were higher for women who had GSM in than those who did not have GSM (Table 3).

**Table 3:** Impact of urogenital symptoms on the different domains of quality of life according to the DIVA questionnaire (n= 285).

Different Domains	Mean±SD
Activities of daily living	0.6±0.7
Emotional wellbeing	1.0±0.8
Sexual functioning (short version)	1.8±1.0
Sexual functioning (long version)^*^	1.5±0.6
Self-concept and body image	1.2±0.7

* calculated in women with sexual activity within last one month

Among all the menopausal women (n= 385), the mean age of the women at consultation was 56.8±7.3 years and the mean age of menopause was 48.2±3.6 years. Most of the women 301 (78.2%) had menopause at or below 50 years of age. The cause of menopause was natural in 351 (91.2%) of cases. Less than half of the women 181 (47.0%) were sexually active, while 316 (82.1%) were married. The majority of the women 227 (59.0%) were illiterate (Table 4).

**Table 4:** Demographic characteristics of the women (n= 385).

Age (years)	n (%)
40-49	44 (11.4)
50-59	211 (54.8)
60-69	92 (23.9)
≥70	38 (9.9)
**Age of menopause (years)**	
≤50	301 (78.2)
>50	84 (21.8)
**Type of menopause**	
Natural	351 (91.2)
Surgical	34 (8.8)
**Marital status**	
Married	316 (82.1)
Widow	62 (16.1)
Divorced/separated	7 (1.8)
Sexually active (within last 1 month)	181 (47.0)
With menopause ≤5years	113 (62.4)
With menopause >5years	68 (37.6)
**Educational status**	
Illiterate	227 (59.0)
Primary	72 (18.7)
Secondary	67 (17.4)
Higher secondary and above	19 (4.9)
**Address**	
Kathmandu valley	263 (68.3)
Outside Kathmandu valley	122 (31.7)
**BMI (kg/m^2^)**	
Underweight	13 (3.4)
Normal	152 (39.5)
Overweight	168 (43.6)
Obese	52 (13.5)

## DISCUSSION

This study was conducted in a tertiary care hospital in Kathmandu. The information was obtained by interview and 227 (59.0%) women were illiterate. The mean age of menopause was 48.2±3.6 years. This is similar to the study done in Nepal among 2000 women where it was 48.7 years.^8^ Other hospital^9-11^ and community based^3,12^ studies of Nepal show the age of menopause ranging from 46 to 49 years and 48 to 51 years respectively.

In the present study, the prevalence of GSM among postmenopausal women was 74.0%. Various cross-sectional studies done in Nepal had shown, among the genitourinary symptoms sexual problems in 47% to 81%, bladder problems in 47% to 57%, and dryness of vagina in 60% to 63%.^3,10,13^ All rating scales increased from pre to postmenopausal women and the severity of the symptoms was higher in post-menopausal.

In the GENISSE study involving 423 Spanish postmenopausal women, the prevalence of GSM was 70.7%.^6^ the prevalence varies as the study population including only postmenopausal women shows higher prevalence than those including premenopausal women as well. In the present study, the urogenital symptoms and signs GSM were more prevalent in women who were menopausal for more than five years in comparison to the lesser duration of menopause.

Among these postmenopausal women, only 181 (47.0%) were sexually active. The sexual activity decreased with increased duration of menopause, where 113 (62.4%) were sexually active with menopause duration within 5 years and only 68 (37.6%) with more than 5 years of menopause. In a study from India, 88.3% out of the 162 postmenopausal women were not sexually active, and GSM was two times more common in women with more than 5 years of menopause, while a study done in Spain showed 72% of postmenopausal women were sexually active.^6,14^ Vulvovaginal atrophy had a significant impact on the ability to be intimate in 62% of women.^15^ Sexual dysfunction may involve decreased interest or desire to initiate activity, as well as decreased arousal or ability to achieve an orgasm during sexual relations. The etiology of sexual dysfunction often is multifactorial, including psychological problems such as depression or anxiety disorders, conflict within the relationship, issues relating to prior physical or sexual abuse, medication use, or physical problems that make sexual activity uncomfortable, such as endometriosis or atrophic vaginitis.^16^

Genital dryness and irritation and burning in the genital are some of the common symptoms in postmenopausal ladies. In the present study, vaginal dryness was the most common symptom affecting 223 (78.2%) in postmenopausal women with GSM. The internet-based survey conducted in Italy, Germany, Spain, and UK on 3768 postmenopausal women showed vaginal dryness in 70%. On analysis of a prospective cohort study over 17 years (1996-2013) from 2435 participants in the Study of Women's Health Across the Nation (SWAN), the prevalence of vaginal dryness increased from 19.4% among all women at baseline (ages 42-53 years) to 34.0% at the thirteenth visit (ages 57-69 years). Advancing menopausal stage, surgical menopause, anxiety, and being married were positively associated with developing vaginal dryness, regardless of partnered sexual activity.^17^ In the present study, almost half of the postmenopausal women with GSM had urinary symptoms, with 137 (48.1%) suffering from dysuria and 156 (54.7%) having urinary frequency/urgency. Two-thirds of women do not relate their vaginal or urinary complaints to menopause,^18^ and they often delay seeking treatment for several years as these symptoms are more often taken as part of aging.

Women with these symptoms had decreased quality of life. In the present study, the vaginal symptoms had the least impact on the activity of daily living, and the highest impact was seen in sexual functioning. There has been a significant effect of GSM on the sexual functioning of women affecting the quality of life.^6,19^ In a survey of 3,046 US women, Real Women's Views of Treatment Options for Menopausal Vaginal Changes (REVIVE), women reported that their vulvovaginal atrophy (VVA) symptoms led to some loss of intimacy (85%), distracted from the enjoyment of sex (59%), interfered with their relationship (47%), negatively affected sleep (29%) and adversely affected general enjoyment of life (27%).^20^ Clinicians can resolve many distressing genitourinary symptoms and improve sexual health and the quality of life of postmenopausal women by educating women about, diagnosing, and appropriately managing GSM.^21^

The present study showed that more than two-thirds of postmenopausal women suffered from genitourinary syndrome when they were assessed by appropriate symptoms and signs. Thus, it is essential to be vigilant on the part of the health workers to keep this aspect of the problem in mind and give appropriate treatment where women would be suffering silently. There may be memory bias especially regarding the age of menopause. The study was hospital-based from Kathmandu valley; thus, the findings of the study may not represent especially in the community setting and that of the country.

## CONCLUSIONS

The study showed three-fourth of postmenopausal women suffered from GSM, which is similar to the studies done in other countries. The study provides information on the prevalence of genitourinary problems after menopause. Vaginal dryness, urinary frequency, and among sexually active women decreased lubrication and pain during contact were the most common symptoms. These symptoms and signs of GSM increases with the duration of menopause. In impact of GSM on quality of life, it least affected the activities of daily living and mostly on sexual functioning. Therefore, understanding GSM is vital, and assuring women that treatments are available to relieve symptoms may improve their quality of life.
